# Small proportions of actively-smoking patrons and high PM_2.5_ levels in southern California tribal casinos: support for smoking bans or designated smoking areas

**DOI:** 10.1186/1471-2458-12-819

**Published:** 2012-09-22

**Authors:** Neil E Klepeis, Jason Omoto, Seow Ling Ong, Harmeena Sahota Omoto, Narinder Dhaliwal

**Affiliations:** 1 Education, Training, and Research, Inc, Scotts Valley, CA, USA; 2 Graduate School of Public Health, San Diego State University Research Foundation, Center for Behavioral Epidemiology and Community Health (CBEACH), San Diego, CA, USA; 3 Department of Civil and Environmental Engineering, Stanford University, Stanford, CA, USA

**Keywords:** Secondhand smoke, Fine particles, Active smoker counts, Nonsmoking gaming

## Abstract

**Background:**

Nearly all California casinos currently allow smoking, which leads to potentially high patron exposure to secondhand tobacco smoke pollutants. Some argue that smoking restrictions or bans would result in a business drop, assuming > 50% of patrons smoke. Evidence in Nevada and responses from the 2008 California tobacco survey refute this assertion. The present study investigates the proportion of active smokers in southern California tribal casinos, as well as occupancy and PM_2.5_ levels in smoking and nonsmoking sections.

**Methods:**

We measured active-smoker and total-patron counts during Friday or Saturday night visits (two per casino) to smoking and nonsmoking gaming areas inside 11 southern California casinos. We counted slot machines and table games in each section, deriving theoretical maximum capacities and occupancy rates. We also measured PM_2.5_ concentrations (or used published levels) in both nonsmoking and smoking areas.

**Results:**

Excluding one casino visit with extremely high occupancy, we counted 24,970 patrons during 21 casino visits of whom 1,737 were actively smoking, for an overall active- smoker proportion of 7.0% and a small range of ~5% across casino visits (minimum of 5% and maximum of 10%). The differences in mean inter-casino active-smoker proportions were not statistically significant. Derived occupancy rates were 24% to 215% in the main (low-stakes) smoking-allowed slot or table areas. No relationship was found between observed active-smoker proportions and occupancy rate. The derived maximum capacities of nonsmoking areas were 1% to 29% of the overall casino capacity (most under 10%) and their observed occupancies were 0.1 to over 3 times that of the main smoking-allowed casino areas. Seven of twelve visits to nonsmoking areas with no separation had occupancy rates greater than main smoking areas. Unenclosed nonsmoking areas don’t substantially protect occupants from PM_2.5_ exposure. Nonsmoking areas encapsulated inside smoking areas or in a separate, but unenclosed, area had PM_2.5_ levels that were 10 to 60 μg/m^3^ and 6 to 23 μg/m^3^ higher than outdoor levels, respectively, indicating contamination from smoking.

**Conclusions:**

Although fewer than roughly 10% of casino patrons are actively smoking on average, these individuals substantially increase PM_2.5_ exposure for all patrons in smoking and unenclosed nonsmoking areas. Nonsmoking areas may be too inconvenient, small, or undesirable to serve a substantial number of nonsmoking patrons. Imposing indoor smoking bans, or contained smoking areas with a maximum capacity of up to 10% of the total patronage, would offer protection from PM_2.5_ exposures for nonsmoking patrons and reduce employee exposures.

## Background

There are currently 63 tribal (Native American) casinos in California, which employ over 50,000 workers, with up to 10 more casinos under construction [1,2]. Close to one third of these casinos are located in populous southern California. Currently, only one tribal casino in California is entirely smoke-free (Lucky Bear Casino). While smoking prohibitions in California went into effect for non-tribal gaming clubs in January 1998, gaming facilities are controlled by tribal sovereign entities and are not subject to California state law unless the law is included in an agreement, known as a “Compact”, between state or local governments and the tribe. As a result, patrons visiting tribal casinos are likely to receive exposure to secondhand tobacco smoke (SHS) pollutants, including airborne fine particles with diameters under 2.5 microns (PM_2.5_). Elevated concentrations of PM_2.5_ are a well-known indicator of the presence of SHS [3], and PM_2.5_ has its own well-established adverse health effects [4,5].

The predominant contribution of smoking to indoor levels of PM_2.5_ and other pollutants in casino air throughout California and elsewhere has been documented by a number of investigators. These studies show clear physical evidence of smoking causing substantial exposure to SHS in both smoking and certain designated nonsmoking areas of casinos. Jiang et al. [6] surveyed PM_2.5_ levels in 36 different California Indian casinos, finding an average concentration of 63 μg/m^3^ in smoking slot areas, a value which was 9 times higher than mean outdoor levels. Nonsmoking areas without complete physical air separation were not protective, with PM_2.5_ levels still reaching, on average, nearly 30 μg/m^3^. York and Lee [7] measured PM_2.5_ in nonsmoking restaurants of 16 Nevada casinos, reporting average levels of 31 μg/m^3^, with average levels of 48 μg/m^3^ in smoking gaming areas. Repace [8] measured particle levels (respirable suspended particles, RSP; and particle-bound polycyclic aromatic hydrocarbons, PPAH) in eight Delaware casinos, finding that the health of casino patrons and staff was endangered -- the casino air contained 20 times more RSP, on average, than outdoor background levels. The RSP and PPAH particles were eliminated by 85% to 95% after the implementation of indoor smoking bans in the Delaware casinos. Repace et al. [9] studied PM_2.5_ in 66 casinos around the US, grouping results from new and prior investigations [6,8,10] and found that indoor (smoking) and outdoor levels were approximately lognormally-distributed with geometric means of 54 and 4 μg/m^3^, respectively. Levels in three nonsmoking casinos averaged 3 μg/m^3^, indicating most or all of the PM_2.5_ in the smoking casinos arose from tobacco smoke emissions. In a study of 17 Australian social and gaming clubs, Cains et al. [11] measured PM_10_ and nicotine in general-use and designated “no smoking areas”. They found that levels in nonsmoking areas could be lower by 50%, but reductions were not as significant as the protection obtained from a complete smoking ban.

Biological measures of casino employees and patrons further demonstrate the effects of smoking in casinos on air quality, health risk, and dose. Urinary cotinine and 4-(methylnitrosamino)-1-(3-pyridyl)-1-butanol (NNAL) are commonly-used biomarkers with sensitivity and specificity for discriminating tobacco smoke exposure [12]. Trout et al. [13] found that casino employees had higher cotinine levels than a representative sample of the US population. Cotinine increased significantly more during the casino work shift than in other work places. Repace [10] found casino patrons had increased cotinine levels. In a recent study of employee exposures in three Las Vegas, Nevada casinos, Achutan et al. [14] performed a survey consisting of biological (urine) samples from 124 dealers for cotinine, total NNAL, and creatinine analysis, and full-shift area and personal breathing zone air samples (n = 113 of the dealers) for nicotine, various volatile organic compounds (VOCs), PAH’s, and RSP. They found average nicotine (geometric mean, GM = 6.7 μg/m^3^) and particle levels (GM = 41 μg/m^3^) similar to what other casino investigators have found. Casino dealers were found, via the biomarker NNAL, to be exposed to a known carcinogen, tobacco-specific nitrosomine 4-(methylnitrosamino)-1-(3-pyridyl)-1-butanone (NNK). Achutan et al., therefore, conclude that the only way to eliminate this specific health risk is to ban smoking in casinos. Anderson et al. [15] found, similarly, that casino patrons excreted NNAL and were, thus, exposed to NNK.

Evidence observed in Nevada casinos indicates that smokers represent a small minority of total casino patrons. Responding to apparent industry claims that smokers comprise more than 50% of all casino patrons, Pritsos et al. [16] measured the density of smokers relative to the total number of gamblers in 18 Nevada casinos in Las Vegas, Reno/Sparks, and Lake Tahoe, implementing a similar methodology to that described by Repace and Lowrey [17]. The results of their study indicated that the overall percentage of active smokers in Nevada casinos was 6.7%, which, when adjusted by a factor of 3 [17], resulted in estimated smoker prevalence of 20%, 22%, and 16% for the Las Vegas, Reno/Sparks, and Lake Tahoe casinos, respectively (20.2% overall). As noted by Pritsos et al. [16], these values are comparable to the reported 2005 smoker prevalence for the total US population of 20.9% [18]. The factor of 3, used by Pritsos et al. and Repace and Lowrey, assumes an average of 2 cigarettes smoked an hour with a cigarette duration of 10 minutes, on average, which results in an average active-smoking period of 20 min per hour or 1/3 of the time. Thus, assuming a random smoking process, cross-sectional measures of the number of active smokers may observe 1/3 of actual smokers, on average.

As noted by Timberlake et al. [19], tribal casinos are of special interest in California, because they represent the last substantial California indoor setting where employees and patrons can be exposed to SHS. Timberlake et al. [19], reporting on the results of the 2008 California Tobacco Survey regarding SHS exposure and SHS avoidance behavior of casino patrons, find that 17.6% of casino patrons smoke and 10.4% of non-patrons are smokers, 60.8% of patrons attempt to avoid SHS by moving about the casino, 67.2% of respondents support a smoking ban, and the likelihood of visiting a casino with physical separation between smoking and nonsmoking areas is associated with avoidance of SHS in never-smokers.

Following the work in Nevada and California by Pritsos et al. [16], Jiang et al. [6], as well as other researchers discussed above, the present study used on-site visits to directly measure the proportion of patrons in southern California tribal casinos, who are actively smoking at any point in time, and thus are responsible for exposures to PM_2.5_ experienced by the bulk of casino employees or other patrons -- in either smoking or nonsmoking areas. Using measures of available gaming machines or tables, as well as original measures of PM_2.5_ in the casinos (and taken from Jiang et al. [6]), we report and discuss the relationship of active-smoker proportions to derived patron occupancy, the relative occupancy of smoking versus nonsmoking areas, and the level of potential protection from PM_2.5_ exposure that is currently offered to casino patrons by nonsmoking areas.

## Methods

### Setting

**Figure 1:**
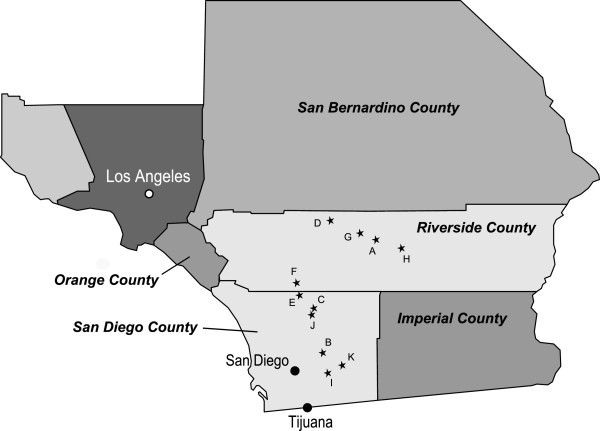
Map of southern California showing geographical locations of 11 casinos visited in the present study.

Over 20 million people live in the six southern California counties surrounding the casinos visited as part of the present study (Los Angeles, Orange, San Diego, Riverside, San Bernardino, and Imperial counties). We selected a convenience sample of 11 Indian Casinos in San Diego and Riverside counties. The selected sites constitute half of a total of 22 casinos in the entire southern California region. We chose casinos that we determined had a smoking-allowed policy and at least one dedicated nonsmoking area, allowing us to measure the proportion of active smokers as well as the occupancy rates and PM_2.5_ levels for smoking and nonsmoking areas. We visited new sites along main thoroughfares until we achieved a sample of casinos that was representative of a wide range of casino types, sizes, smoking/nonsmoking area characteristics and geographic locations. Casino size ranged from 9,144 sq m (30,000 sq ft) to 198,120 sq m (650,000 square ft). See Table 1 and Figure 1, respectively, for a summary of casino characteristics and a map of the 11 casino sites, with each casino assigned an identifying letter (A through K).

**Table 1:** Characteristics of each casino visited in the present study and dates of count visits

**Casino**^**a**^	**Square Footage**^**b**^	**Total Number of Slot Machines Counted**^**c**^	**Total Number of Table Games Counted**^**c**^	**Number of Tables in Poker Area**^**c**^	**Number of High Stakes Slot Machines**^**c**^	**NS Area Type**^**c,d**^	**Dates of Count Visits**^f^
**A**	70,000	1900	38	11	50	Open	10/24; 10/25
**B**^**e**^	310,000	2230	50	15	30	Open	8/23; 8/30
**C**	55,000	1585	54	15	85	Open	10/10; 10/11
**D**	150,000	2030	103	21	100	Open	10/24; 10/25
**E**	650,000	2200	76	N/A	50	Enclosed	10/26; 11/1
**F**	188,000	2100	287	55	250	Open	10/17; 10/18
**G**	30,000	1120	26	8	20	Open	10/25; 10/26
**H**	110,000	1900	44	8	20	Partial	10/24; 10/25
**I**^**e**^	305,000	2570	83	23	20	Enclosed	9/5; 9/6
**J**	62,000	1614	18	N/A	114	Partial	10/10; 10/11
**K**	210,000	2540	60	18	40	Partial	8/16; 8/23

^a^Casino names have been replaced with anonymous identifiers.

^b^Data on square footage for each casino was obtained from the 500 Nations Website [1].

^c^Counts of slots and tables were determined from the on-site (firsthand) observations made in each casino for the present study in both nonsmoking (NS) and smoking (S) areas. The type of nonsmoking area for each casino was also observed firsthand for the present study.

^d^“Enclosed” nonsmoking (NS) areas were defined as being in a different room that had enclosing walls limiting direct air flow from smoking (S) areas; “Partial” (partially separate) nonsmoking areas were in a different room but had no barriers to air flow from the smoking areas; “Open” nonsmoking areas were in the same room and not physically separated from the main casino smoking areas. See Table 5 for further notes on the type of nonsmoking area observed in each casino.

^e^Square footage values for Casinos B and I were not available on 500 Nations Website [1] and were obtained from other online sources including casino-specific websites (not cited here to avoid casino disclosure).

^f^Date of casino visits where counts of patrons were made, two per casino, in MM/DD format. All visits were made on Friday or Saturday evenings or early the next morning (from 6 pm to 3 am) in 2008. The time difference between the two visits ranged from 1 day to 7 days. See Table 5 for the dates on which we made PM_2.5_ measurements.

Following the designation of Jiang et al. [6], we defined three types of nonsmoking areas in each casino: (1) “Open” areas that had no separation from smoking areas, i.e., were in the same room, perhaps in a corner or a roped-off area; (2) “Partial” (i.e., partially separate) areas that were in a separate room but had no apparent barriers to prevent air from travelling through doorways or hallways; and (3) “Enclosed” areas that were in a separate room from smoking areas with a closable door to prevent direct smoke transfer.

### Materials

During each casino visit, each of two investigators used a standard 4-digit hand tally counter to record either the number of actively-smoking patrons or total number of guests in the casinos. Data were transferred to an observation sheet via paper and pencil. In four of the casinos, we measured 1-minute airborne fine particle mass concentrations (PM_2.5_) using an AM510 SidePak Personal Aerosol Monitor (TSI Inc., Shoreview, MN). The SidePak was configured with a 2.5-micron impactor and the air flow was set to 1.7 LPM before each monitoring visit. A custom calibration factor of 0.29 for indoor SHS was determined for the SidePak unit used in the present study by Jiang et al. [6], who performed experimental filter-and-pump gravimetric calibration of a number of SidePak units in an automobile cabin. A field SidePak calibration factor for indoor SHS was determined by Jiang et al. [20] in a northern California casino in a rural setting (with smoking) to be 0.29, thereby corroborating the experimental result and justifying use of the 0.29 factor inside casinos where SHS is likely to be the predominant aerosol. We used a single SHS calibration factor of 0.29 for all indoor and outdoor SidePak measurements. Due to practical considerations, we did not determine or apply an outdoor calibration factor for each casino. Outdoor levels were expected to be very low and, therefore, relatively insensitive to adjustment factors in absolute value. Based on the work of others, calibration factors for outdoor aerosol, unlike those for SHS, can vary on a day-to-day basis at the same location. For example, Jiang et al. [20] determined calibration factors of roughly 0.3 and 0.5 for outdoor air near a rural casino, with and without wood smoke, respectively, and a median factor of 0.77 (standard deviation, 0.14) for outdoor aerosol in two outdoor urban locations. Zhu et al. [21] found a factor of 0.3 for outdoor aerosol, close to the factor for SHS, using controlled chamber experiments.

### Dimensions and count observation

We performed counting visits to each of the 11 casinos between August and November 2008 on Friday or Saturday evenings (or sometimes early the next morning) between 6 pm and 3 am. Each casino was visited on two separate occasions with observation intervals ranging from 30 min to 120 min depending on the size of the casino. The time between visits to a given casino was 1 to 7 days. All observations were accomplished by two investigators (one counting total patrons and one counting active smokers), except for Casino I, in which a single investigator performed all the counts. All observations were carried out covertly using concealed hand counters. Each casino was divided into up to six different sections: (1) smoking high-stake slots, (2) smoking main area slots, (3) smoking high-stakes table games, (4) smoking main area table games; (5) poker rooms, and (6) nonsmoking sections, which generally contained slots. *Main areas* that allowed smoking had low-stakes slot or table games and occupied most of the casino gaming area (see Table 2). Two casinos (Casinos E and J) did not have a poker room on the premises and Casinos C, H, and J did not have high-stakes table games.

**Table 2:** **Estimated gaming capacity by casino area**^**a**^

**Casino**	**Estimated Total Gaming Capacity [persons]**	**Estimated Main Area S Gaming Capacity**^**b**^**[persons]**	**Estimated NS Gaming Capacity**^**c**^**[persons]**	**Proportion of NS Capacity to Total Gaming Capacity**	**Ratio of NS Gaming Capacity to Main Area S Gaming Capacity**
**A**	2238	1942	100	4.5%	5.1%
**B**	2680	2168	200	7.5%	9.2%
**C**	2069	1724	110	5.3%	6.4%
**D**	2858	2500	30	1.0%	1.2%
**E**	2656	2420	150	5.6%	6.2%
**F**	4372	2320	100	2.3%	4.3%
**G**	1356	1144	100	7.4%	8.7%
**H**	2244	2064	80	3.6%	3.9%
**I**	3298	2378	610	18.5%	25.7%
**J**	1722	1108	500	29.0%	45.1%
**K**	3080	2288	500	16.2%	21.9%

^a^Total smoking (S) and/or nonsmoking (NS) capacity estimated assuming maximum of 6 patrons per game table, 10 patrons per poker table, and 1 patron per slot machine. The number of slots, table games, and poker tables were determined by our on-site (firsthand) observations in the present study.

^b^Main Area capacity refers to smoking (S) low-stakes slot machines and table games (i.e., excluding poker tables and high stakes slots). The Main Area gaming capacity (low stakes slots + tables) is a quantity used in the present work to calculate relative occupancy between nonsmoking and smoking areas. The occupancy for non-main smoking areas (poker or high-stakes slots/tables) can be calculated by subtracting the Main Area capacity from the Total capacity.

^c^Except for Casino I, which had 10 table games, nonsmoking capacity refers to the number of slot machines in nonsmoking areas.

Upon arriving at a casino, investigators first performed an initial walk-through to measure the approximate dimensions of each section, the number of slots and game tables in each section, and a rough impression of the occupancy and number of smokers in each section of the casino (approximate proportion of seats filled and proportion of people who were smoking) to insure the casino was substantially occupied with smokers and nonsmokers (i.e., more than approximately 1/3 full). The casino floor dimensions were measured by counting the number of footsteps taken by the observer over the width and length of the various sections of each casino. Subsequently, the investigators walked around the entire casino, performing a formal count of the number of smokers and total number of guests in each section of the casino. The first observer counted the total number of people within a 3 m radius (10 ft), while the second observer counted the total number of smokers. A smoker was defined as a person actively holding or puffing a lit cigarette, cigar, or tobacco pipe in his or her hand, or having one of the previously mentioned tobacco instruments lit in an ashtray within 0.30 m (1 ft) of the person. Persons with an unlit cigarette, cigar, or tobacco pipe within 0.30 m of the person were not considered to be actively smoking (e.g., a pack of cigarettes exposed from a woman’s purse was not counted). Due to the size of the casinos, and the time needed to count all individuals, it is possible that certain individuals were counted twice. However, since movement of people is likely random, this effect was not expected to impact our results. The observers walked side-by-side approximately 0.30 meters (1 foot) apart from each other down each row of game tables or slot machines until the entire section was counted.

### PM_2.5_ observation

Using the same methodology as Jiang et al. [6], we performed PM_2.5_ (fine particle) air monitoring in four casinos (Casinos F, H, I and J) between December 2010 and October 2011. The PM_2.5_ measurements were made on different days than the count observations were made, i.e., PM_2.5_ was not contemporaneous with counts, but they were made on generally the same days of the week and times (early or late evenings on Fridays or Saturdays, or early the next morning). During monitoring, investigators concealed the SidePak in a purse, handbag, or coat pocket -- with inlet tubing sticking out a number of inches -- and behaved like normal patrons, e.g., played slot machines. Sequential 1-minute measurements were taken of outdoor PM_2.5_ concentrations for a period of ~10 minutes, followed by monitoring time periods of 20 or 30 minutes in each of the main smoking gaming area of the casino and the nonsmoking gaming area of the casino, and, finally, a second ~10-minute outdoor monitoring period. This approach allowed for direct comparison of PM_2.5_ in the three basic sub-settings of each casino (outdoor, smoking, nonsmoking). We report data from Jiang et al. [6] on outdoor, smoking-area, and nonsmoking-area PM_2.5_ in the remaining seven casinos under study in the present work (casinos A, B, C, D, E, G and K). The Jiang et al. visits occurred between February and March 2008, the same year as our own counting visits, and, except for one visit, took place on Friday or Saturday evenings – i.e., the same general time frame as our count measurements.

### Data analysis

Statistical testing for the difference between two proportions was used in analyzing the count data. For each casino, the mean active-smoker proportion and standard deviations were determined. ANOVA was used to test the difference between the count means with a p < 0.05 significance level.

To provide a standard basis for quantifying the degree of occupancy across casinos, we derive and report a *theoretical gaming capacity* of smoking and nonsmoking gaming areas based on our on-site (firsthand) accounts of the number of slots, tables games, and poker tables that were present, and assuming 1 maximum person per slot machine, 6 maximum persons per table game, and 10 persons per poker table. This quantity indicates the theoretical maximum number of people that can be engaged in gaming at any point in time. We define and report the observed *occupancy rate* as the number of observed persons in a given area of the casino divided by that area’s theoretical gaming capacity. The occupancy rate can exceed 100% if more people are present in the area than the maximum that could theoretically be engaged in gaming behavior, e.g. instead of gaming they may be observing others, socializing, or simply passing by. During our second visit to Casino F, a conference that was being held resulted in over 8000 total patrons. The conference may have influenced the proportion of active smokers, and we therefore omitted the data from this visit in our analysis but still provide the individual Casino F results.

We analyzed the PM_2.5_ air monitoring data by taking averages of 1-minute readings across total time spent outdoors, in the smoking area, or in the nonsmoking area of each casino. To provide a comparative measure of different casino sub-settings, we calculated the difference in the average concentrations of the smoking and nonsmoking areas, and of the nonsmoking area and the outdoors.

## Results

In Table 1, we present the observed characteristics for each casino, including the raw number of tables and slot machines counted, and the type of nonsmoking area. During our visit, six of the casinos had “open” nonsmoking areas with no separation from smoking areas (i.e., they were in the same room), three casinos had areas that were “partially separate” from smoking areas in a separate room (but with no apparent air barrier), and two casinos had “enclosed” nonsmoking areas that were separated from smoking areas by a closed door (Casinos E and I). Table 2 contains the estimated theoretical gaming capacity for smoking and nonsmoking areas. The capacity of nonsmoking areas was typically a small fraction of the overall gaming capacity of each casino (1% to 29%; 8 casinos under 10%). In Tables 3 and 4, we present, respectively, the observed patron and active-smoker counts: (1) across all smoking and nonsmoking gaming areas (broken out by visit); and (2) in smoking-allowed gaming areas only (high stakes and main area slots; and high stakes and main area table games). *Main smoking areas* with slot and table games had low-stakes wagers and occupied most of the casino gaming capacity (Table 2). For all except one visit, we did not observe any active smokers in poker rooms. In 6 of 21 visits, active smokers were observed in the nonsmoking (non-poker) areas. We totaled combined counts across the two visits for each casino to provide average derived quantities (Table 3). Overall, we counted 24,970 patrons across 21 visits at 11 tribal casinos, with 1,737 who were actively smoking, for an overall *active-smoker proportion* of 6.96%, excluding the second visit to Casino F for which the casino was highly-occupied at 3.6 times the patron occupancy of the first visit. The mean active-smoker proportion between casinos did not show any significant differences (F = 0.394 (10), p = 0.94).

**Table 3:** **Patron and active-smoker counts, and occupancy and active-smoker proportions for all areas by casino visit**^**a**^

**Casino**	**Visit 1**				**Visit 2**					
	**Total Patrons**	**Smokers**	**% Active Smokers**	**% Occupancy**	**Total Patrons**	**Smokers**	**% Active Smokers**	**% Occupancy**	**Overall Mean (% Active Smokers)**	**Mean Difference between Visits (% Active Smokers; Visit 2 – Visit 1)**
A	1133	78	6.88%	50.6%	1082	85	7.86%	48.3%	7.36%	0.97%
B	1234	71	5.75%	46.0%	1141	93	8.15%	42.6%	6.91%	2.40%
C	819	58	7.08%	39.6%	945	72	7.62%	45.7%	7.37%	0.54%
D	1126	78	6.93%	39.4%	1820	93	5.11%	63.7%	5.80%	−1.82%
E	1195	74	6.19%	45.0%	1318	97	7.36%	49.6%	6.80%	1.17%
F^b^	2360	210	8.90%	54.0%	**8576**	**159**	**1.85%**	**196.2%**	**3.37%**	**−7.04%**
G	607	49	8.07%	44.8%	457	37	8.10%	33.7%	8.08%	0.02%
H	862	83	9.63%	38.4%	929	85	9.15%	41.4%	9.38%	−0.48%
I	1451	86	5.93%	44.0%	1309	72	5.50%	39.7%	5.72%	−0.43%
J	1006	61	6.06%	58.4%	1228	84	6.84%	71.3%	6.49%	0.78%
K	1107	69	6.23%	35.9%	1841	102	5.54%	59.8%	5.80%	−0.69%
Overall^b^	12900	917	7.11%	--	12070	820	6.79%	*--*	*6.96%*	*0.93%*^*c*^

^a^Data shown are for the entire casino, i.e., for the total smoking and nonsmoking areas. All occupancy rates and active-smoker proportions areas are expressed as a percentage (% Occupancy;% Active Smokers). Occupancy is the number of total patrons divided by the theoretical capacity of all smoking areas. Smoker-activity as a function of occupancy for main smoking areas is presented in Figure 2. Occupancy for nonsmoking areas (slots + tables) relative to main smoking areas is presented in Figure 3.

^b^During the second visit to Casino F, patron occupancy was substantially higher than the first visit, resulting in a decrease in active smoker count proportion. We considered this event to cause unlikely deviations and therefore excluded it from our overall analysis. It is, however, presented here in disaggregated format for reference. The overall row contains the row-wise sums for the Patrons and Smokers columns, and the overall percentages across all casinos for a given visit or across all visits. Data for the overall row excludes all the results from the second visit to Casino F (numbers in bold).

^c^The overall mean difference in the last column excludes the second visit to Casino F (number in bold), and considers only the magnitude of difference between visits, not the direction.

**Table 4:** **Patron and active-smoker counts and active-smoker proportions for smoking table and slot games by casino**^**a**^

	**Table Games – Main Area**			**Table Games – High Stakes**^**b**^			**Slot Machines – Main Area**			**Slot Machines – High Stakes**		
**Casino**	**Total Patrons**	**Smokers**	**% Active Smokers**	**Total Patrons**	**Smokers**	**% Active Smokers**	**Total Patrons**	**Smokers**	**% Active Smokers**	**Total Patrons**	**Smokers**	**% Active Smokers**
A	440	23	5.23	22	1	4.55	1440	136	9.44	25	1	4.00
B	640	38	5.94	75	7	9.33	1217	111	9.12	23	6	26.09
C	428	31	7.24	-	-	-	1087	93	8.56	26	3	11.54
D	744	50	6.72	14	1	7.14	1746	113	6.47	78	7	8.97
E	470	43	9.15	24	1	4.17	1708	126	7.38	9	1	11.11
F^c^	319	33	10.35	145	5	3.45	1518	163	10.74	43	9	20.93
G	193	8	4.15	5	1	20.00	755	74	9.80	16	2	12.50
H	250	23	9.20	-	-	-	1527	145	9.50	1	0	0.00
I	389	31	7.97	13	1	7.69	1827	121	6.62	17	5	29.41
J	123	5	4.07	-	-	-	1480	136	9.19	78	4	5.13
K	373	21	5.63	64	5	7.81	1246	121	9.71	101	16	15.84
TOTAL	4369	306	7.00	362	22	6.08	15551	1339	8.61	417	54	12.95

^a^Data shown are for smoking-allowed areas only and combine both visits to each casino. All active-smoker proportions for smoking areas expressed as percentages (% Active Smokers). Main smoking area slot or table areas had low-stake games and occupied most of each casino’s gaming capacity (see Table 2).

^b^Entries with a dash (“-”) indicate that no separate game tables were present in the corresponding casino.

^c^The second visit to Casino F is excluded from the data in this table. On the day of this visit, we noted that the patron occupancy was much higher than the first visit, resulting in an occupancy that was 3.6 times that on the first visit and a decrease in active smoker counts by 7%. All the per-visit data for Casino F, and all the other casinos, are presented in Table 3.

Excluding the second visit to Casino F, absolute differences between the two visits averaged 0.93%, ranging from 0.02 to 2.4%, with 7 of 10 casinos below 1% (Table 3). The visit with the lowest proportion had 5.1% active smokers (Casino D) and the visit with the highest proportion had 9.6% (Casino H). The active-smoker proportion averaged across both visits to each casino ranged from 5.7% to 9.4%.

Across the different types of gaming areas that allowed smoking (Table 4), the highest observed active-smoker proportions were for high-stakes slot machines (average of 13%). However, high-stakes areas did not have a large number of patrons in most of the casinos. Three of the casinos (C, H, and J) did not offer high stakes for table games. The overall mean active-smoker proportion for slot machines (8.61%) and table games (7.00%) in the main smoking areas were similar. The differences between the mean active-smoker proportion for these four gaming areas were marginally statistically significant (F = 2.804 (3), p = 0.053).

### Active-smoker proportion relative to occupancy

**Figure 2:**
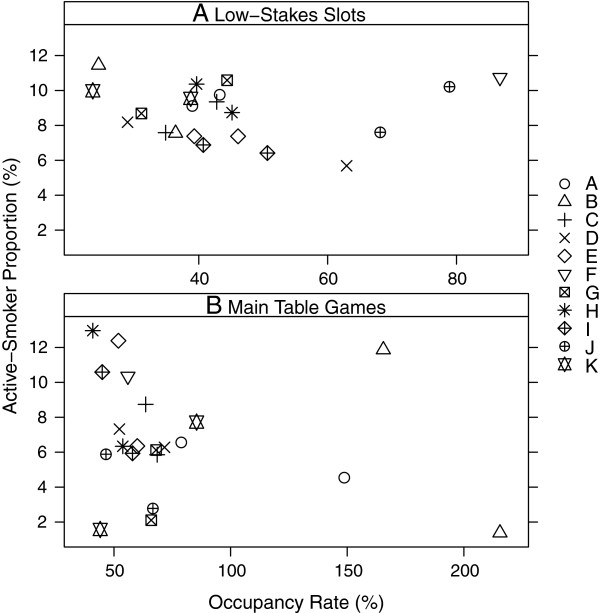
(**A and B). The relationship between observed active-smoker proportion and occupancy rate in main smoking areas (low-stakes tables and slot**s). [(**A**) low-stakes smoking slots, and (**B**) low-stakes smoking tables. The proportion of active smokers, expressed as a percent, is calculated as 100 x observed active smokers divided by observed total people, and occupancy rate is calculated as 100 x observed total people divided by theoretical maximum capacity. Two separate visits to each casino are shown in the plots using matched plotting symbols. Results shown exclude the second visit to Casino F in which a very large occupancy was observed].

To study the change in active-smoker proportion as a function of occupancy, we plotted observed active-smoker proportions versus the theoretical occupancy rate for individual visits to the 11 casinos (Figure 2). Only main smoking areas (low stakes slot machines and table games) were included, because the largest sample of patrons was observed in the main areas and including the low number of patrons in the high-stakes areas gives rise to highly-skewed distributions of active-smoker proportion. For slot machines in the main low-stakes area (excluding second visit to Casino F), for 17 of 21 visits the casinos were occupied at or below 50% (range of 24% to 86%,), while for the table games the estimated occupancy was above 50% for 17 of 21 visits (range of 40% to 215%). For both table games and slot machines, active-smoker proportions stayed fairly stable relative to the occupancy rate. We observed a larger range between casino visits for the game tables (1% to 13% active-smoker proportions) compared to slot machine areas (6% to 11.5%) – both excluding the second visit to Casino F.

### Occupancy of nonsmoking areas

**Figure 3:**
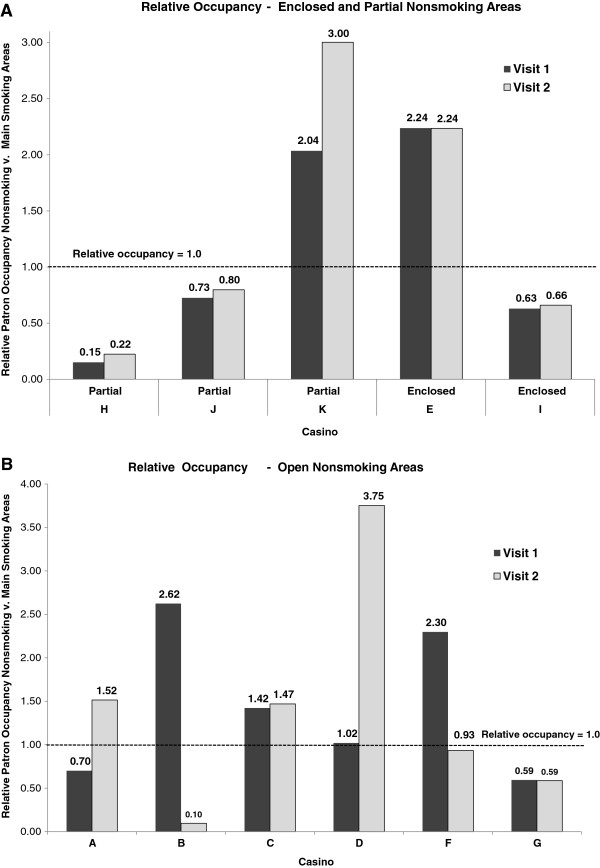
**(A and B). The occupancy of patrons in nonsmoking areas relative to the main (low stakes) smoking areas.** [(**A**) casinos with partially separate or enclosed nonsmoking areas (i.e., having enclosing walls and a door); and (**B**) casinos with open nonsmoking areas. The bars for each casino show results for the two separate visits. Relative occupancy was calculated as the occupancy rate (observed total people divided by theoretical maximum capacity) within the nonsmoking areas (slots + tables) divided by the occupancy rate in the main smoking-allowed areas of the casino (low-stakes slots + tables). A relative occupancy of 1.0 (ratio) indicates that the occupancy rate of the nonsmoking area is equal to that of the main smoking areas; less than or greater than 1 indicates that the nonsmoking area is proportionally less or more occupied relative to the main gaming areas].

Each of the casinos we visited had a designated nonsmoking area, although characteristics of these areas varied (see definitions of “open”, “partially separate”, and “enclosed” designations given above). For each visit, we divided the occupancy rate of all slot and table games in nonsmoking areas by the occupancy rate of the main gambling areas with smoking (low-stakes slots and tables) to obtain a *relative occupancy rate* – broken down by whether nonsmoking areas were enclosed or partially-separated versus completely open to the smoking areas (Figure 3). The relative occupancy quantity has a value of 1 when the nonsmoking area and main smoking areas have the same occupancy rate.

Both visits to 3 of 5 casinos with partially-separate or fully-enclosed nonsmoking areas had occupancies that were 15% to 80% of the main smoking areas (main low-stakes slots and table games) (Figure 3A). On both visits, the enclosed nonsmoking section at Casino E had slightly more than double the occupancy of the main smoking areas. Casino K had a partially-separate nonsmoking occupancy that was 2 to 3 times the main smoking area occupancy. Casino E had an enclosed nonsmoking room that was located within 30 m (100 ft) of the main entrance and contained tables and slot machines, and may have presented a convenient and attractive location for nonsmokers to spend time. Similarly, Casino K had a large, partially-separate nonsmoking area with easy access. While Casino I, like Casino E, had an enclosed nonsmoking game room with tables and machines, including a separate nonsmoking entrance, it was not occupied at a higher rate than main areas. Neither was Casino J’s large, partially-separate nonsmoking area with tables, slots, and a bar occupied at higher rate. Here, one had to walk through the casino smoking section to reach the nonsmoking section. Casino H had a similar nonsmoking section with a partially-separate slot machine room, but did not have tables or a bar located in the room and had a very low relative occupancy rate (15% to 22% of the main smoking areas).

There was more variation apparent in relative occupancies for nonsmoking areas in casinos with open nonsmoking areas, which were located within or immediately adjacent to smoking areas, versus those in casinos with other types of nonsmoking areas (Figure 3B). Five of the 6 casinos with open nonsmoking areas had at least one visit in which relative occupancy was greater than 1 (for 7 of the 12 total visits). These casinos typically displayed signs stating “This area is a nonsmoking area” and did not have ashtrays near the slot machines, or the nonsmoking areas may have had red ropes to indicate the area perimeter to guests. However, the smoke emitted in smoking areas was not ostensibly prevented from freely entering the nonsmoking area, although unseen barriers such as an air curtain may have been present. Casino G was the only casino with an open nonsmoking area that had nonsmoking relative occupancies less than 1 for both visits, but this was a small casino with a nonsmoking gaming capacity that was only 7% of the total (Table 2). For all of the other 10 casino visits to open nonsmoking areas, relative occupancy was between 1 and 3.75 for 7 visits and under 1 for only 3 visits. While there was a fully-enclosed nonsmoking room in Casino D, it was closed during both of our observation visits. A staff member of the casino reported that the casino had decided to keep the doors of the nonsmoking room locked because it did not have enough volume and the casino did not wish to pay taxes on the unused slot machines. On our visits to Casino D, we observed an open nonsmoking area.

### Air quality in smoking and nonsmoking areas

The average PM_2.5_ concentrations in each of the 11 visited casinos (measured on separate days from when counting was performed), observed either by Jiang et al. [6] or from original measurements in the current study, ranged from 44 to 110 μg/m^3^ in main smoking areas (Table 5). For comparison, the current U.S. EPA 24-hour standard for outdoor PM_2.5_ levels is 35 μg/m^3^[22]. Concentrations in nonsmoking areas were more than 25 μg/m^3^ lower than those in smoking areas, except for two casinos that had open nonsmoking areas (Casinos C and F) with nearly the same concentration as smoking areas (5.2 μg/m^3^ lower and 1.5 μg/m^3^ higher, respectively). Enclosed nonsmoking areas offered strong protection from PM_2.5_ relative to smoking areas (Casinos E and I) with reductions of 66 and 78 μg/m^3^. Partially-separate nonsmoking areas, including those that were partially-separate during PM_2.5_ measurement and later apparently changed to an open area (Casinos B, D, H, and J; no data available for K), offered reductions of 37 to 64 μg/m^3^. The partially-separate or enclosed nonsmoking areas had levels 2 to 23 μg/m^3^ higher than the clean outdoor levels, indicating their inability to eliminate elevated PM_2.5_ exposures. The open nonsmoking areas in Casinos C and F had PM_2.5_ levels ~60 μg/m^3^ above clean outdoor levels, clearly showing the inadequacy of these areas to protect nonsmokers from high PM_2.5_ exposures due to smoking. Based on others’ laboratory and field work [20,21], we expect the SidePak outdoor aerosol calibration factor for rural areas, which was not determined in this study, to be reasonably comparable to the SHS factor applied for all indoor levels where SHS is likely the predominant particle source. Even considering larger factors by 2/3, the absolute PM_2.5_ levels for outdoor air measured here would only increase by 2.2 μg/m^3^, on average. Therefore, use of the same calibration factor for indoor SHS and outdoor (ambient) aerosol is reasonable, and we do not expect that much error is introduced.

**Table 5:** **Mean PM**_**2.5 **_**concentrations measured in smoking and nonsmoking casino areas and the outdoors**

		**PM**_**2.5 **_**Concentration,**^**c**^** μg/m**^**3**^					
**Casino**^**a**^	**Type of Nonsmoking Area**^**b**^	**Smoking Area, S**	**Nonsmoking Area, NS**	**S-NS Difference**	**Outdoors**^**c**^	**NS-Outdoors Difference**	**Date of Visit**^**f**^
A	Open	94	32	62	2.4	30	2/09/08
B	Open (Partial)^d^	74	10	64	4.2	5.6	3/28/08
C	Open	62	57	5.2	1.3	56	3/29/08
D	Open (Partial)^e^	76	25	51	1.3	23	2/09/08
E	Enclosed	72	6.5^c^	66	4.4	2.1	3/30/08
F*	Open	61	62	−1.5	4.7	58	10/15/11
G	Open	44	17	28	6.7	9.8	2/11/08
H*	Partial	57	20	37	2.9	17	10/15/11
I*	Enclosed	86	8^c^	78	1.9	6.1	12/11/10
J*	Partial	50	11	39	1.3	9.9	1/22/11
K	Partial	110	-	-	5.3	-	3/28/08

^a^For all casinos not marked with an asterisk, PM_2.5_ concentrations are the results of Jiang et al. [6]. For the casinos marked with an asterisk, PM_2.5_ concentrations were measured as part of the present study.

^b^“Enclosed” nonsmoking (NS) areas were in a different room that had enclosing walls limiting direct air flow from smoking (S) areas; “Partial” (partially separate) NS areas were in a different room but had no barriers to air flow from the smoking areas; “Open” NS areas were in the same room and not physically separated from the main casino smoking areas.

^c^A SidePak calibration factor of 0.29 was used for the original PM_2.5_ indoor measurements in Casinos F, H, I, and J (smoking or nonsmoking). This factor for SHS was determined through a controlled car-cabin (indoor) test and also corroborated with a field test inside a casino [20]. It is expected that the factor applies to all indoor locations, since SHS is likely the predominant aerosol in both smoking and nonsmoking areas. For outdoor aerosol, the factor’s value is assumed to be comparable. based on the work of others [20,21], but could be higher depending on conditions. Regardless, since the absolute outdoor concentrations are small, any resulting error is expected to be low. For example, with an increase in the factor of 2/3, the concentrations would average 5.5 instead of 3.3 μg/m^3^, an increase of only 2.2 μg/m^3^.

^d^The Jiang et al. [6] study designated Casino B NS area as Partial, but on our trip it was determined to be Open. During our visit, the NS area was on the main floor off to the side of the main smoking area in a corner of the casino.

^e^Casino D has a separate NS area that was closed during our visit but was visited by Jiang et al. [6].

^f^Date of visit is given on which PM_2.5_ was measured in MM/DD/YY format. All visits took place on Friday or Saturday early or late evenings or in the early morning hours of the following day, except for Casino G, which was visited in the early evening on a Monday.

## Discussion

Similar to Pritsos et al. [16], we found that the percentage of patrons actively smoking at any given time (active-smoker proportion) was very consistent from casino-to-casino with no significant differences. The overall average value reported by Pritsos et al. [16] for Nevada of 6.74% was quite close to our reported overall value of 6.96% for southern California casinos. Jiang et al. [6] and Repace et al. [9] found somewhat higher mean values of 11% and 10% for active-smoker proportions in California-wide tribal casinos and Reno, NV casinos, respectively.

Although the present study does not provide direct data on the actual percentage of patrons who are smokers (the *smoking rate*), following Pritsos et al. [16] and Repace and Lowrey [17] (also see Repace [23]), a rough estimate can be obtained by multiplying active smoker proportions by 3. When we multiply our overall estimate of active-smoker proportion by 3 (6.96% x 3), we obtain an estimated smoking rate of 21%. Pritsos et al. [16] found an average smoking rate in Nevada casinos of 20%. Thus, the results in the present study are consistent with the findings of Pritsos et al., which indicate the proportion of smokers in casinos roughly mirrors that of the smokers in the US population as a whole [16]. However, according to the most recent Census data available by county, the adult smoking rate for Riverside and San Diego counties was 12.7% and 11.0%, respectively [24]. In addition, Timberlake et al. [19], utilizing data from the 2008 Adult California Tobacco Survey, report a smoking prevalence of 17.6% for casinos patrons and 10.4% for non-patrons. Hence, the proportion of smokers in southern California casinos may be closer to double that of the regional smoking rate – although still representing fewer than Â¼ of patrons.

Our results indicate that the number of nonsmokers + non-active smokers outnumbers the number of active smokers by an average ratio of 13 to 1. Even considering that the average apparent enrichment of smokers in the measured casinos is at a rate of approximately double that of the general population in southern California, nonsmokers will still outnumber smokers by roughly 4 to 1.

The work of Timberlake et al. [19] suggests many existing patrons would welcome smoking bans, or avenues to reduce SHS exposure, with more than 90% of respondents saying their stay duration would increase or remain unchanged in the face of a smoking ban. Our results are consistent with efforts to protect nonsmoking patrons (the majority) from the secondhand smoke generated by smoking patrons (the minority) – either by instituting smoking bans or establishing a separated enclosed or removed location in which smoking can occur.

The levels of PM_2.5_ we observed are comparable to what others have found, indicating substantial exposure to secondhand smoke, including for known toxic compounds (e.g., PPAH) and carcinogens (e.g., NNK). Currently, the number of nonsmokers, who are exposed to secondhand smoke, exceeds those that can seek refuge in nonsmoking areas. However, many casino owners may not be ready to institute total smoking bans. Another option, the expansion of nonsmoking areas, would generally not result in adequate protection from PM_2.5_ exposures, and nonsmokers may also continue to occupy easy-to-access areas where smoking is allowed and/or ones they perceive as having better machines and services or more lively action. A better and more effective approach to eliminating hazards of secondhand smoke exposure (in the absence of a total smoking ban) may be to designate indoor or outdoor smoking areas that could accommodate up to approximately 10% of the total casino patronage at a given time (consistent with our estimates of the maximum number of actively-smoking patrons across the casinos we visited). This approach would give a minority of patrons a place to smoke and leave the majority of the casino entirely smoke free. With an indoor smoking area, employees would generally be likely to experience lower SHS exposures, although those working in the smoking area would remain exposed. As recommended by Wagner et al. [25], to minimize leakage, indoor smoking areas should be depressurized with respect to adjoining nonsmoking areas and they should use a sliding door as opposed to a swinging-type door.

### Limitations of this study

The count data presented in this study are focused on times when casinos were more than approximately 1/3 full and for a clientele makeup that is likely to be present in casinos on weekend evenings (Friday and/or Saturdays). The main limitation of the PM_2.5_ measurements presented in this study is their time separation relative to the measurement of person counts. This time separation limits the ability to directly link number of observed active smokers with PM_2.5_ levels.

## Conclusions

Consistent with previous research on smoking in casinos cited above, the results of the present study indicate that the proportion of actively-smoking patrons is consistently a small percentage of the total number of guests (overall average of 7.0% with per-casino values of less than 10%) with occupancy rate having no apparent influence on the proportion. The actual number of people who smoke at least one cigarette in the casino may be closer to 20%. However, this minority activity apparently dictates the overall casino smoking policies and PM_2.5_ exposures for all patrons. Nonsmoking areas provided by the casino are generally too small to accommodate all nonsmokers and/or they are not used by nonsmokers (perhaps due to inconvenience or lower attractiveness). Furthermore, nonsmoking areas that were not fully enclosed generally offered insufficient protection for nonsmokers to PM_2.5_ levels caused by smoking. A better approach, which would insure the protection of nonsmokers and also accommodate smokers, would be to establish separate indoor (enclosed) or outdoor smoking areas that could handle up to ~10% of the maximum casino occupancy, which is the likely maximum proportion of active smokers at a given time.

## Abbreviations

NNAL: 4-(methylnitrosamino)-1-(3-pyridyl)-1-butanol [urinary metabolite of NNK, known lung carcinogen]; NNK: 4-(methylnitrosamino)-1-(3-pyridyl)-1-butanone [tobacco-specific carcinogen]; PM_2.5_: Airborne particulate matter with particle diameter under 2.5 microns; PPAH: Particle-bound Polycyclic Aromatic Hydrocarbons; RSP: Respirable Suspended Particles; SHS: Secondhand tobacco Smoke; VOC: Volatile Organic Compound; GM: Geometric Mean.

## Competing interests

NEK works primarily as a secondhand smoke exposure researcher in academia where he is funded by a variety of private, state, and federal health agencies. He also works as a paid consultant on secondhand smoke-related projects, and occasionally as a legal expert witness on secondhand smoke issues. SLO and ND work on projects funded by tobacco-related policy organizations, such as the California Department of Public Health. The authors declare no other competing interests.

## Authors’ contributions

NEK designed the study protocols, oversaw data analysis, helped with graphic preparation, and led drafting of the manuscript. JO performed field data collection and helped draft the manuscript. SLO performed data analysis and helped draft the manuscript. HSO performed field data collection. ND initiated the study concept, participated in the study design, coordinated the study, performed field data collection, and assisted with drafting the manuscript. All authors read and approved the final manuscript.

## Authors’ information

NEK holds a Doctorate in Environmental Health Science and has performed secondhand smoke exposure and behavior research for nearly 20 years. He has contributed seminal studies on secondhand smoke outdoors, in residences, and in vehicles. JO is currently working towards a Masters of Science in Applied Behavior Analysis. SLO holds a Masters in Social Work and works as a research associate and data manager on school health and tobacco control projects, providing quantitative and qualitative analysis methods, as well as database management. HSO holds a Masters in Multicultural Education and currently works as a 5^th^ grade teacher. ND holds a Masters in Gerontology and is the project director of California’s Clean Air Project (CCAP), a project funded by the California Department of Public Health that provides technical assistance, training, and educational materials related to the issue of secondhand smoke.

## Pre-publication history

The pre-publication history for this paper can be accessed here:

http://www.biomedcentral.com/1471-2458/12/819/prepub
